# Antigenic Fingerprinting following Primary RSV Infection in Young Children Identifies Novel Antigenic Sites and Reveals Unlinked Evolution of Human Antibody Repertoires to Fusion and Attachment Glycoproteins

**DOI:** 10.1371/journal.ppat.1005554

**Published:** 2016-04-21

**Authors:** Sandra Fuentes, Elizabeth M. Coyle, Judy Beeler, Hana Golding, Surender Khurana

**Affiliations:** Division of Viral Products, Center for Biologics Evaluation and Research (CBER), FDA, Silver Spring, Maryland, United States of America; University of Chicago, UNITED STATES

## Abstract

Respiratory Syncytial Virus (RSV) is the major cause of pneumonia among infants. Here we elucidated the antibody repertoire following primary RSV infection and traced its evolution through adolescence and adulthood. Whole genome-fragment phage display libraries (GFPDL) expressing linear and conformational epitopes in the RSV fusion protein (F) and attachment protein (G) were used for unbiased epitope profiling of infant sera prior to and following RSV infection. F-GFPDL analyses demonstrated modest changes in the anti-F epitope repertoires post-RSV infection, while G-GFPDL analyses revealed 100-fold increase in number of bound phages. The G-reactive epitopes spanned the N- and C-terminus of the G ectodomain, along with increased reactivity to the central conserved domain (CCD). Panels of F and G antigenic sites were synthesized to evaluate sera from young children (<2 yr), adolescents (14–18 yr) and adults (30–45 yr) in SPR real-time kinetics assays. A steady increase in RSV-F epitope repertoires from young children to adults was observed using peptides and F proteins. Importantly, several novel epitopes were identified in pre-fusion F and an immunodominant epitope in the F-p27. In all age groups, antibody binding to pre-fusion F was 2–3 folds higher than to post-fusion form. For RSV-G, antibody responses were high following early RSV infection in children, but declined significantly in adults, using either G proteins or peptides. This study identified unlinked evolution of anti-F and anti G responses and supportive evidence for immune pressure driven evolution of RSV-G. These findings could help development of effective countermeasures including vaccines.

## Author Summary

Respiratory syncytial virus (RSV) is the major cause of pneumonia and bronchiolitis among infants and children globally. In the United States, RSV infections lead to 57,000 hospitalizations among young children, especially in those less than one year old. Furthermore, despite the development of immunity following RSV infection during childhood, individuals remain susceptible to RSV upper respiratory tract reinfection. In the current study we explored the antibody repertoires following primary RSV infection and their evolution in adolescents and adults. Whole genome-fragment phage display libraries (GFPDL) expressing linear and conformational epitopes from RSV fusion protein (F) and attachment protein (G) were used for unbiased epitope profiling of sera prior to and following RSV infection. In addition, Plasmon Surface Resonance (SPR) was used to measure antibody binding to F and G peptides and proteins. A steady increase in RSV-F epitope repertoires from young children to adults was observed. Several novel epitopes were identified in pre-fusion F and an immunodominant epitope in F0-p27. For RSV-G, antibody responses were high following RSV infection in children, but declined in adults. This study identified unlinked evolution of anti-F and anti G responses that could help development of better RSV vaccines and therapies.

## Introduction

Respiratory syncytial virus (RSV) is the major cause of pneumonia and bronchiolitis among infants and children globally[1]. In the United States, RSV infections lead to 57,000 hospitalizations among young children, especially in those less than one year old [2]. Many of these infections occur in the presence of passively transferred maternal antibodies, although high titers of neutralizing antibodies appear to ameliorate the disease process in infants less than 9 months of age [3] [4, 5]. Furthermore, despite the development of immunity following RSV infection during childhood, individuals remain susceptible to RSV upper respiratory tract reinfection life-long [4, 6, 7].

RSV isolates can be classified into two antigenically distinct groups (A and B) with genetic differences occurring most extensively in the attachment glycoprotein G (47%) and to a lesser degree in the fusion protein F (9%) [8]. In addition, continuous evolution of subgroup A RSV generates diversity primarily in the G gene [9, 10]. Repeated RSV infections occur mainly with heterologous strains and to a lesser degree with homologous RSV strains due to more restricted diversity [11, 12].

Multiple studies over three decades have explored antibody responses before and following RSV infection in different age groups (infants, children, adults, and older populations). The methods used varied among labs (virus neutralization; antibody binding to infected cells, competition with mouse MAbs against F and G proteins; binding to short peptides and proteins by ELISA). However, few conclusions were reached regarding the best correlates of protection, the reasons for recurrence of RSV infections throughout life, and the evolution of RSV G gene in circulating strains [13–15] [16–25]. Although the importance of RSV as a respiratory pathogen has been recognized for over 50 years, a vaccine is not yet available because of several problems inherent in RSV vaccine development. A better in-depth understanding of the humoral immune responses to primary RSV infection in young children can provide important information that could help design effective countermeasures including vaccine for this age group and for maternal vaccination. We have previously used whole genome fragment-phage-display libraries (GFPDL) spanning the entire genome of highly pathogenic avian influenza virus (HPAI) H5N1-A/Vietnam/1203/2004 to map the antibody repertoires of convalescent sera from H5N1 infected individuals [26].

In the current study, we generated GFPDL for the RSV surface proteins F and G to elucidate the complete antibody epitope repertoire in serum samples from infants either prior (<9 months) or after primary and early RSV infection (15–18 months). Phage display libraries were constructed separately for RSV-F and RSV-G genes to display protein segments ranging in size between 15–250 amino acids and thus predicted to present all possible linear and conformational epitopes for both F and G proteins. Based on the results of the GFPDL epitope repertoire analysis we synthesized large peptides representing the antigenic sites spanning the RSV-F and G proteins and used real-time Surface Plasmon Resonance (SPR) based kinetic analysis with panels of serum from different age groups (<2 yr; 10–14 yr; 30–45 yr) to delineate the evolution of the antibody responses. These approaches identified predominant epitopes recognized by antibodies acquired following primary and early RSV infection and across different age groups. Our study provides comprehensive antigenic fingerprinting of the antibody responses to RSV-F and G proteins following early RSV infection. We have identified several novel epitopes in RSV F and G proteins and an immunodominant epitope in the F-p27, which is excised during processing of F0. Our studies demonstrated an unlinked evolution of the antibody responses to F and G proteins in humans.

## Results

### Generation and characterization of the F and G genome fragment phage display libraries (GFPDL)

**Fig 1:**
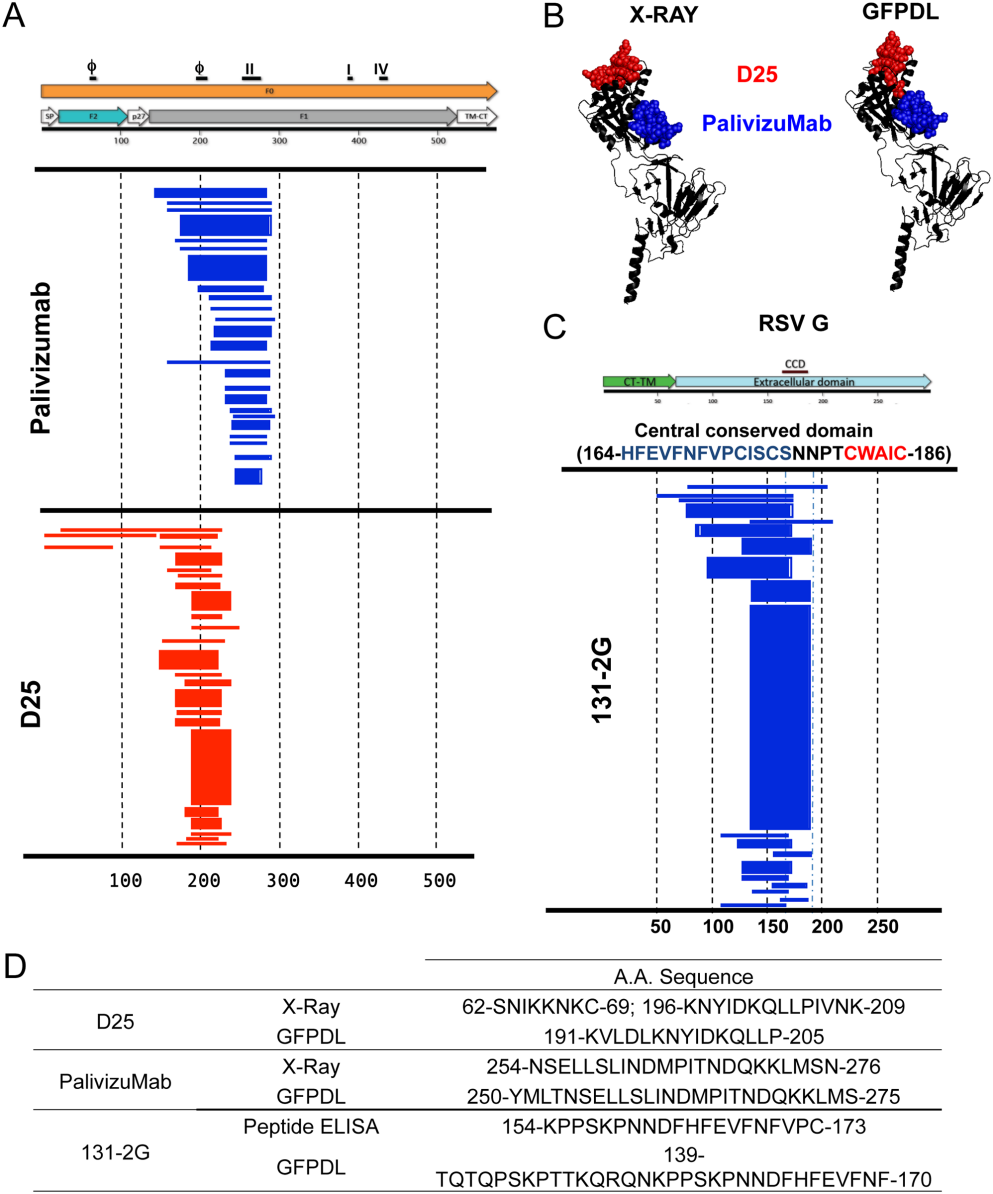
Evaluation of RSV F and G gene fragment phage display library using neutralizing monoclonal antibodies. (A) A gene fragment library displaying random fragments and spanning the entire F gene was used to map the binding epitope of anti-F monoclonal antibodies, Palivizumab and D25. The schematic on top represents the F protein including the location of previously discovered antigenic regions: ϕ, I, II and IV. The alignments of the displayed fragments on the phages selected by Palivizumab and D25 in the F-GFPDL are shown in blue and red bars, respectively. The thickness of the bars represents the relative frequencies of the bound fragments. Numbers at the bottom represent the amino acid residues of the RSV-F protein. (B) Representation of the Palivizumab and D25 antibody epitopes identified by either X-Ray crystallography or GFPDL within the pre-fusion F protein structure (PDB Id- 4JHW). Red refers to the D25 epitope and blue represents the Palivizumab epitope. (C) A gene fragment library displaying random fragments and spanning the entire G gene was used to map the binding epitope of MAb 131-2G. Schematic represents the G protein including previously identified central conserved domain (CCD). (D) Consensus amino acid sequences identified as the epitope recognized by the monoclonal antibodies using GFPDL or other methods.

To analyze the epitope repertoire of serum samples from RSV-infected individuals, GFPDL containing fragments of the F or G genes ranging from 50–750 bp in length were generated with >10^6^ unique phage clones, that are expected to display all possible linear and conformational epitopes. Sequencing of the RSV-F and RSV-G GFPD libraries confirmed random distribution of size and sequence of inserts and spanned the entire F or G genes. To confirm the potential of the phage display libraries to identify both linear and conformational antibody epitopes, we used a panel of 16 anti-F and anti-G MAbs for epitope mapping using the RSV-F and G GFPDL, respectively. Representative GFPDL epitope analysis for two anti-F MAbs and one anti-G MAb is shown in Fig 1. D25 is a conformation-dependent monoclonal antibody that binds site ϕ on pre-fusion form of RSV-F [27]. Palivizumab is the only FDA-licensed product for prevention of RSV infection in high-risk infants. Palivizumab, is a humanized monoclonal antibody that binds to a predominantly linear epitope within antigenic site II of the F protein, which is exposed on both pre-fusion and post-fusion forms of F [28]. Panning of the F-GFPDL with Palivizumab or D25 MAbs identified fragments with sequences that encompassed site II (Palivizumab) or site ϕ (D25) respectively (Fig 1A). The consensus immunodominant epitope sequences for the monoclonal antibodies obtained through GFPDL analysis were very similar to the major binding sites identified by X-ray crystallography (Fig 1B). Mapping of MAb D25-selected specific phages displayed epitope encompassing the complete 62–209 sequence that contains the two contact sites identified by X-ray crystallography studies (Fig 1B). Monoclonal antibody 131-2G binds to an epitope close to the RSV G central conserved domain (CCD)[29]. Affinity selection using RSV-G GFPDL with MAb 131-2G identified multiple fragments, all containing the complete or part of the central conserved domain (Fig 1C).

The minimal common epitope sequences for the binding sites of these MAbs on the F and G proteins identified by the GFPDL selection were compared with their footprints previously identified by either X-ray crystallography or by peptide scanning (Fig 1D). The good agreement between the epitopes identified by GFPDL and the previously identified target sequences for these and additional MAbs provided important proof-of-concept in support of using the RSV F- and G- GFPDLs for elucidating both linear and conformation-dependent epitopes of MAbs and for mapping antibody epitope profiles in human polyclonal sera.

### Mapping of antibody epitope repertoire in children following primary RSV infection

RSV infection occurs very early in life and provides only partial immunity, and although the likelihood of lower respiratory tract infection diminishes with each subsequent exposure, upper respiratory tract re-infection with RSV is common throughout life [5]. To study antibody responses following primary and early RSV infection in an unbiased way, RSV GFPD libraries were used to determine the epitope repertoires of polyclonal serum antibodies against the RSV fusion protein F and attachment protein G.

In a preliminary study, we evaluated the capacity of the GFPDLs to react with hyperimmune sera from rabbits vaccinated three times with RSV-F (post-fusion form) or RSV-G[30] proteins from RSV-A2 (S1 Fig). Pre-vaccination sera did not react with the F-GFPDL, confirming lack of nonspecific binding. Post-fusion F protein vaccinated sera bound large number of phages displaying epitopes that mapped to F1 site II, the C-terminus of F1 and to a dominant site in the C-terminus of F2 (S1A and S1B Fig). Similarly, pre-vaccination sera did not bind to the G-GFPDL, while the post G-vaccination sera reacted with short and large fragments either upstream or downstream of the central conserved domain (CCD) (S1C Fig).

We next determined the capacity of the GFPDLs to adsorb RSV specific antibodies in the rabbit sera. After two rounds of adsorption with the GFPDLs, 86–88% of anti-F antibodies and 89% of anti-G antibodies in the post-vaccination rabbit sera were adsorbed by the F- and G-phage display libraries, respectively, as determined by binding to F and G proteins using SPR. Together, the early studies with rabbit immune sera (and pre-vaccination control sera) provided support for using the RSV F-and G-GFPDL to dissect the polyclonal antibody repertoires in human sera.

To evaluate humoral immune response following primary RSV infection in humans, five children with paired-sera collected prior to infection (at 9 months) and following primary/early RSV infection (prior to 15–18 months) were selected for mapping of antibody epitope repertoires. The infection status was determined by luciferase immunoprecipitation system (LIPS-N), which measures antibodies against the RSV nucleoprotein (N) [positive RSV infection >8000 renilla light units (RLU)], and by plaque reduction neutralization test (PRNT) as previously reported [31] (S1 Table). In addition, isotype profiles of the antibodies demonstrated significant fractions of IgA antibodies against F and G proteins, ranging between 12–37% of total antibodies (S2 Fig), confirming that the 15/18 month sera were acquired post-RSV infection. Anti-RSV maternal antibodies that crossed the placenta could persist up to 9 months, as previously reported [13, 15, 32].

**Fig 2:**
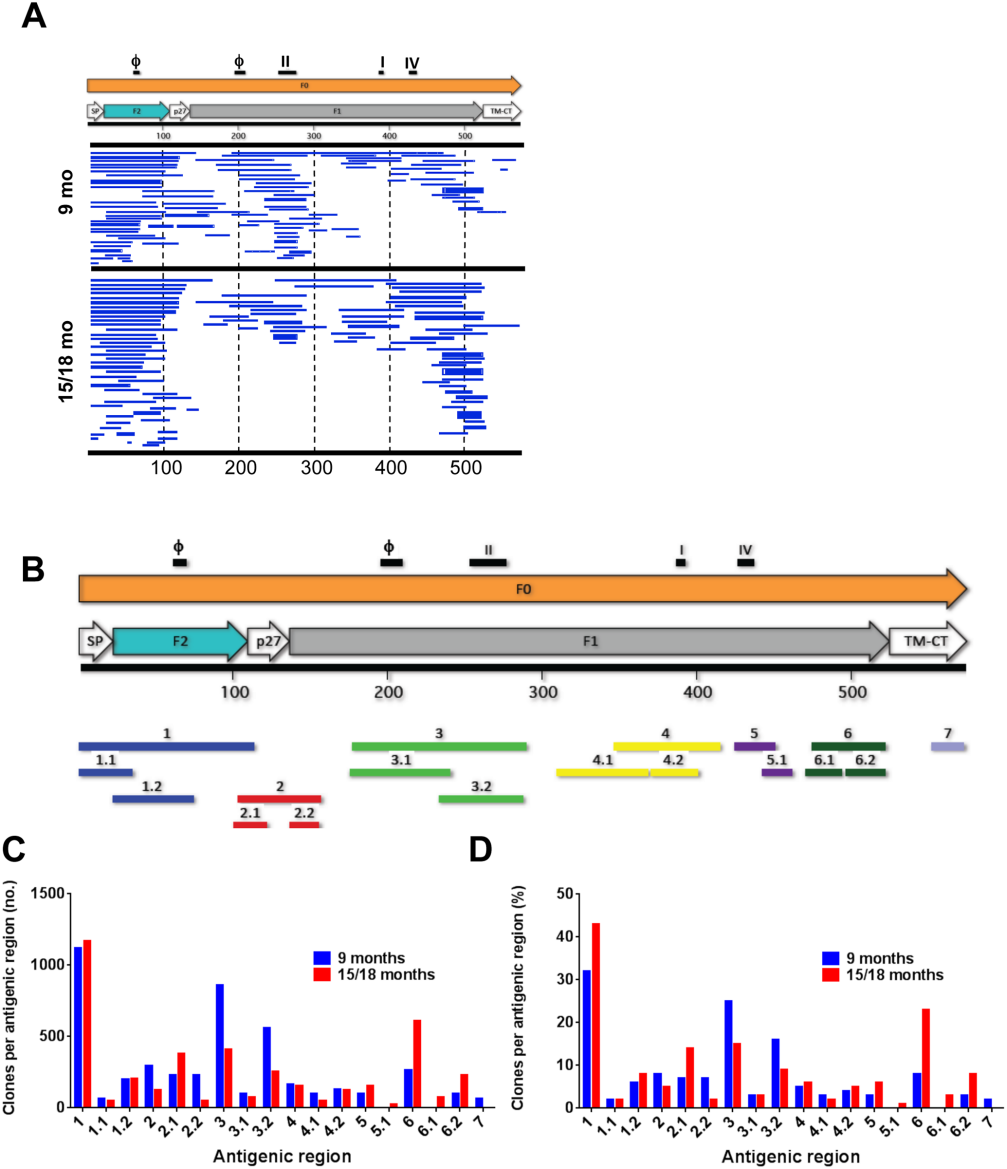
Elucidation of antibody epitope profile against the RSV F protein following RSV primary infection in children. (A) Distribution of phage clones after RSV-F GFPDL affinity selection with sera obtained from children at 9 months (top) or 15–18 months of age (bottom). The amino acid designation is based on the RSV-F protein sequence. Bar location indicates the homology of the displayed RSV-F protein sequence on the phage clones after affinity selection. The thickness of each bar represents the frequencies of repetitively isolated phage inserts (only clones with a frequency of two or more are shown). (B) Antigenic sites within the RSV F protein recognized following primary RSV infection. Previously described antigenic sites (sites ϕ, I, II and IV) are shown above, and the antigenic regions discovered in this study are depicted below the F protein schematic, and are color coded. (C) The number of clones that encoded for each antigenic site before and after RSV infection. (D) Distribution and frequency of phage clones expressing each of the key RSV F antigenic sites. The number of clones that encoded for each antigenic site was divided by the total number of F-GFPDL clones and represented as a percentage before and after RSV infection.

Pooled sera from five children that were negative for anti RSV-N antibodies and RSV neutralizing activity at 9 months of age and seroconverted by 15–18 months were used for F-GFPDL panning (Fig 2). Surprisingly, there was no significant increase in the number of F-GFPDL phages bound by post-RSV infection pooled sera compared with pre-infection sera (Fig 2A). Since our GFPDL panning uses protein A/G to capture the phage-bound antibodies we probably missed some contributions by IgA antibodies to antibody epitope profile in the post RSV infection sera. Furthermore, the contribution of maternal antibodies to the total anti- F antibodies in the plasma from 15–18 months old children was significantly lower than at ≤ 9 months, but we could not separate maternal vs. infant IgG antibodies in the GFPDL analysis. The pre- and post-infection polyclonal sera showed similar antibody epitope repertoires. High frequency of bound phages displayed fragments mapping to the N-terminus of the RSV-F protein that encompasses the F2 subunit and site ϕ, as well as to antigenic site II, and the C-terminus of F (amino acid 400–550) (Fig 2A). Eighteen unique antigenic sites defined by 7 large antigenic regions (herein called F-1 to F-7) and 11 smaller antigenic sites (herein called F-1.1 to F-6.2) were identified in F protein that included both known and novel epitopes (Fig 2B). The numbers of phage clones that were bound by the 9 months vs. 15–18 months sera to each antigenic site are shown in Fig 2C and percentage of clones bound to each antigenic site are shown in Fig 2D. Similar distribution was observed with only few peptides recognized more frequently by the 15–18 month post- RSV infection children sera.

**Fig 3:**
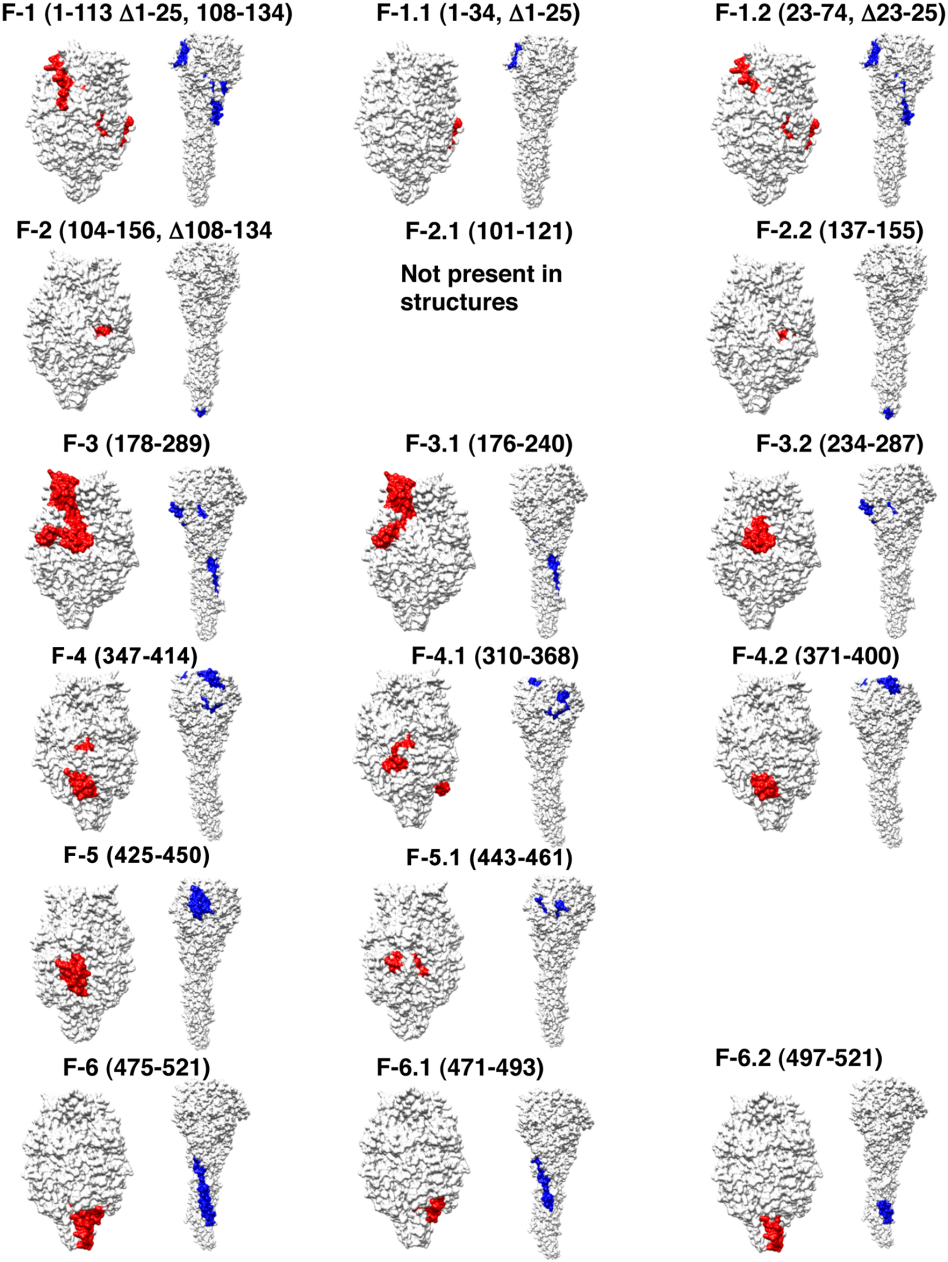
Structural representation of antigenic sites in pre- and post-fusion form of F identified using GFPDL. The surface structure of the pre-fusion trimeric form of RSV F protein (PDB Id—4ZYP) is shown in white and the antigenic region in a monomer (chain A) is highlighted in red. The surface structure of the RSV F post-fusion trimer (PDB Id—3RRT) is shown in white and the antigenic regions from one monomer are highlighted in blue. The RSV F protein used for crystallography encompasses amino acid residues 26-513del108-134 of the complete 574 a.a. long protein sequence. In addition, amino acids 98–109 are not represented in the structures.

The surface exposure locations of each of these antigenic sites in the pre-fusion and post-fusion forms of F are shown in Fig 3. The majorities of the key antibody targets identified by F-GFPDL analysis of post-RSV infection sera are exposed on the native F structures, and are likely to be represented on RSV virions. Two novel antigenic sites (F2, F3), specific for pre-fusion form of F protein were identified that have not been described before (Fig 3 red vs. blue sites on pre-fusion vs. post-fusion F, respectively. Based on the surface representation, antigenic sites 2 and 3 are more accessible in the pre-fusion form of F protein compared to the post-fusion structure. Antigenic site 4 is closer to membrane in the pre-fusion F and may have lesser accessibility in the pre-fusion form of F, but more readily exposed on the crown of the post-fusion F protein (Fig 3).

Most of the epitopes preferentially bound by post-infection sera mapped to F-1, F2.1, and to the C-terminus of the protein (antigenic site F-6). The antigenic sites F-1 (part of site ϕ), F-3 (containing sites ϕ and II) and F-3.2 (site II) were recognized by both pre-and post-infection sera (Fig 2C blue vs. red bars). These data suggested that primary RSV infection in the face of pre-existing maternal antibodies did not induce a very strong anti-F antibody response. But some shift in the predominant epitope recognized following primary RSV infection was identified with increased binding to the N- and C-termini of the F protein, which includes site ϕ, site IV, and the membrane proximal domain.

**Fig 4:**
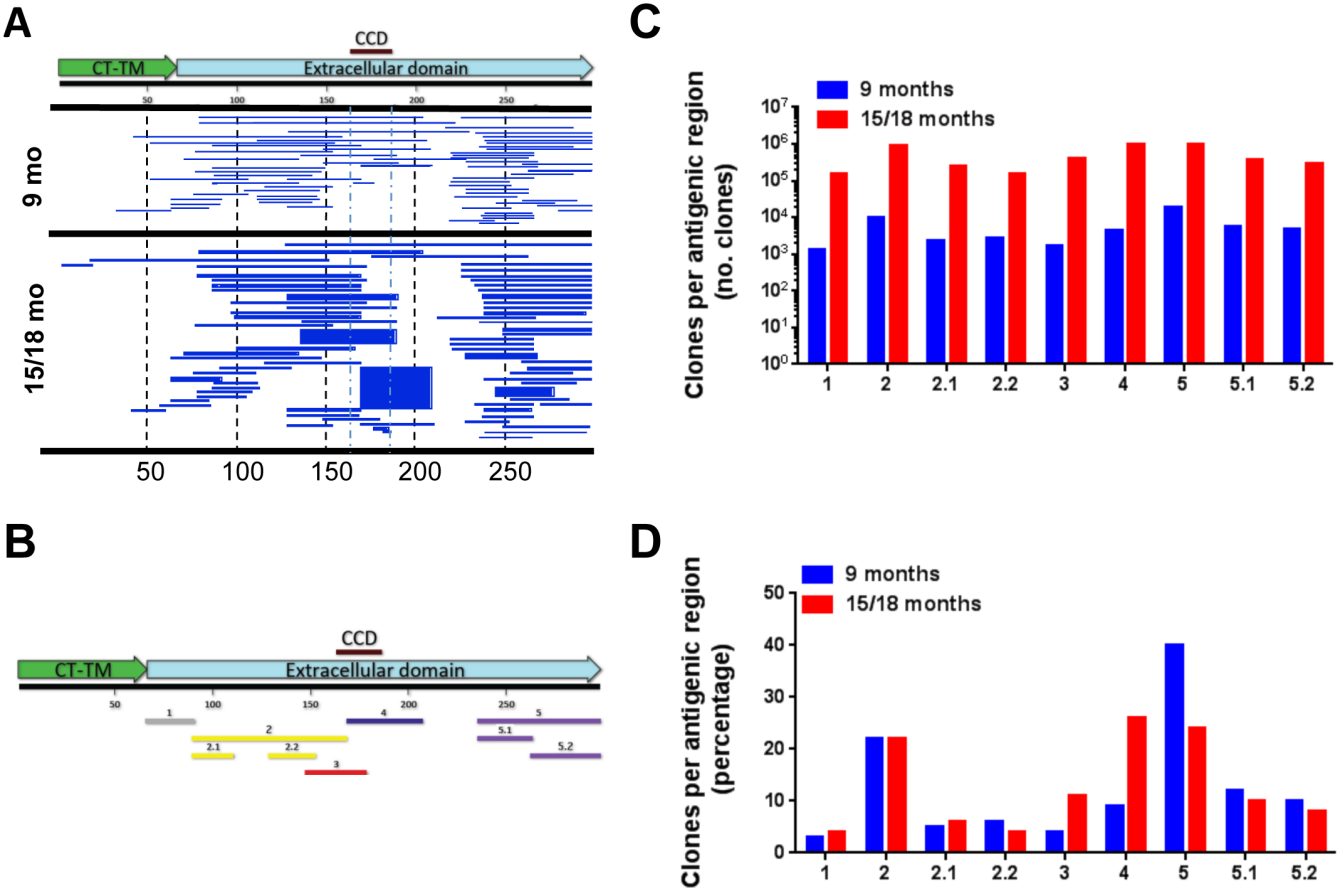
Elucidation of antibody epitope profile against the RSV G protein following RSV primary infection in children. (A) Distribution of phage clones after RSV-G GFPDL affinity selection with sera obtained from children at 9 months (top) or 15–18 months of age (bottom). The amino acid designation is based on the RSV-G protein sequence. Bar location indicates identified inserts of the phage clones after affinity selection mapped on the RSV-G protein. The thickness of each bar represents the frequencies of repetitively isolated phage inserts (only clones with a frequency of two or more are shown). (B) Antigenic sites within the RSV G protein identified following primary RSV infection using GFPDL analysis are depicted below the G protein and are color coded. CCD motif is shown on top of the F protein schematic. (C) The number of clones that encoded for each antigenic site before and after RSV infection. (D) Frequency and distribution of the antigenic regions are represented as a percentage of the total number of affinity selected phage clones. The number of clones that code for each antigenic site was divided by the total number of phage clones and represented as a percentage before and after RSV infection.

In contrast to the modest change in anti-F antibody titers and epitope repertoire, analysis of anti-G antibodies, using the G-GFPDL, revealed a 100-fold increase in the number of bound phages by post-RSV infection sera compared with pre-infection sera (Fig 4A and 4C). The bound phages displayed epitopes that spanned most of the ectodomain of RSV-G with two large possibly conformational regions flanking the CCD motif (Fig 4A). The five main antigenic sites (herein called G-1 to G-5) containing nine epitope regions including some novel epitopes identified through panning of the RSV-G GFPD library are shown in Fig 4B. The post-RSV infection sera selected higher numbers of bound phages with inserts covering all of the identified G antigenic sites (Fig 4C). However, a larger proportion of phages enriched by post-RSV infection antibodies preferentially bound the RSV-G CCD (antigenic site G-4) and a sequence N-terminal to the CCD (antigenic site G-3) compared with pre-infection sera (Fig 4D red vs. blue bars).

Taken together, these results demonstrate that primary infection with RSV induces a strong boost in anti-G antibody levels. Interestingly, the highly conserved CCD region was not a major target of residual maternal antibodies, but became a predominant target of post- RSV infection serum antibodies.

### Antibody binding kinetics to key antigenic sites in RSV F and G proteins with human sera from children, adolescents, and adults

Selected peptides of RSV-F and G proteins representing the key antigenic sites identified by F-GFPDL (Fig 2) and G-GFPDL (Fig 4) were chemically synthesized and tested for antibody binding of individual serum samples as measured by resonance units (RU) in a real-time SPR antibody kinetics experiment. In SPR, both IgG and IgA antibodies contribute to binding. However bias toward dimeric IgA over IgG binding is avoided by ensuring that spatial density of the proteins on the chips is optimized for single monovalent interaction for each antibody molecule irrespective of the isotype.

**Fig 5:**
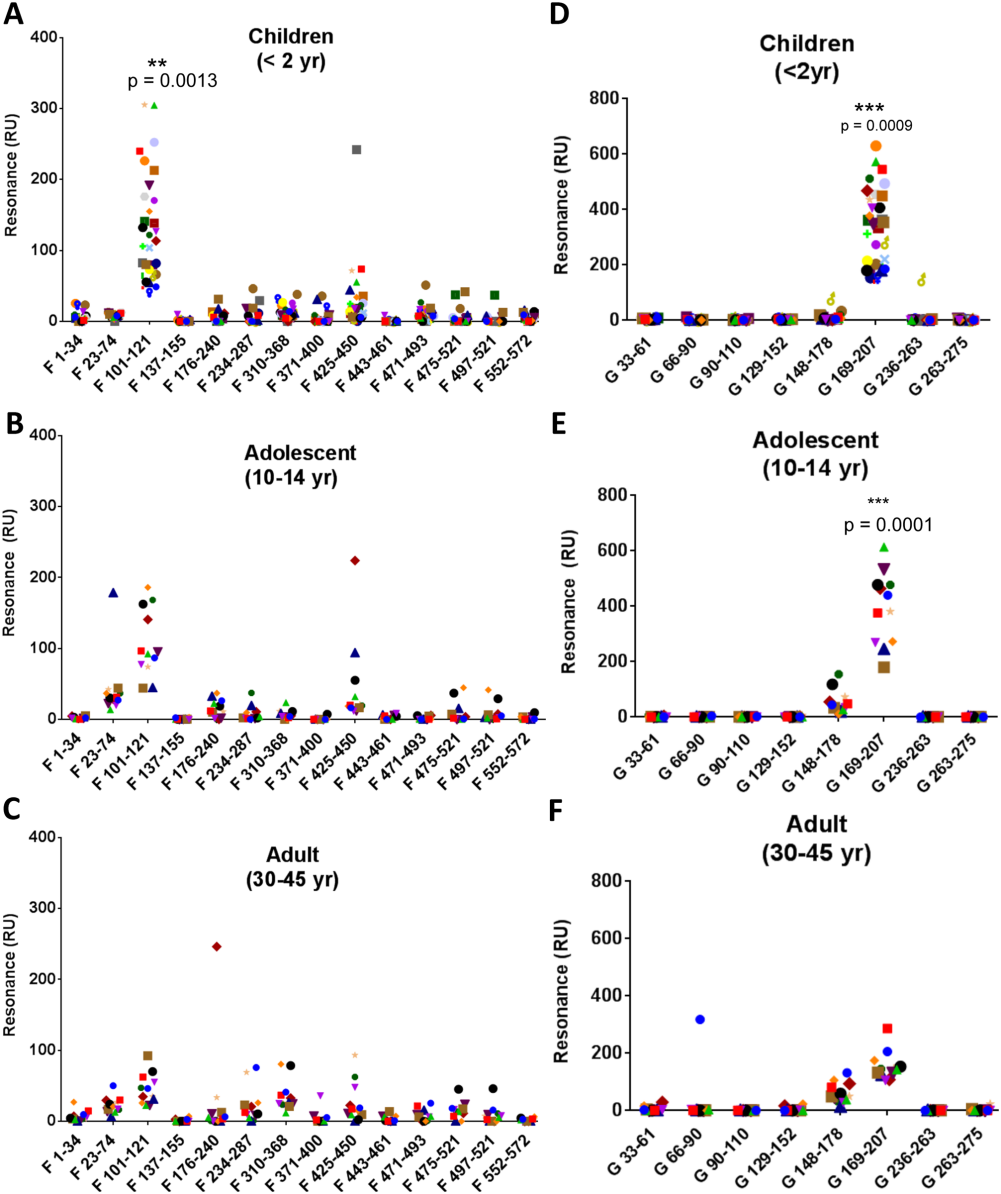
Surface plasmon resonance (SPR) analysis of human sera from of different age group for binding to peptides from different antigenic sites within RSV-F and RSV-G. Selected peptides of RSV-F and G proteins representing the antigenic sites in Figs 2 and 4 were chemically synthesized and tested for binding against individual sera samples using real time SPR kinetics experiment. Total antibody binding is represented as SPR resonance units (RU). Panels A-C show total antibody binding against the F peptides and panels D-F show total antibody binding to G peptides with serum/plasma samples from children less than 2 years old (A and D), adolescents; 10–14 years old (B and E) and adults 30–45 years old (C and F). Peptides in the X-axis are labeled by RSV-A2-F subunit followed by amino acid sequence (A-C) and for RSV-A2-G protein followed by amino acid sequence (D-F). Statistical significant differences of antibody reactivity between different age groups are depicted with *p* values.

Individual serum samples from children [less than 2 years old (N = 30)], adolescent [10–14 years old (N = 12)], and adult [30–45 years old (N = 12)] were evaluated (Fig 5 and S2 Table). All sera from very young children (<2yr) (100%) reacted with a novel antigenic site F-2.1 (aa 101–121) that maps to p27 and is uniquely found only in the uncleaved F0 protein (mean RU = 130), and 58% sera reacted with a antigenic site F-5 peptide (aa 425–450) (mean RU = 25). Reactivity to all the other F peptides ranged between 0%-35% with low mean RU values (Fig 5A and S2 Table). All the samples in this age group were also tested in the PRNT to differentiate between those that were most likely to be post-infection (positive titers) vs. uninfected children (no neutralizing antibodies) (S3A and S3B Fig). The peptide binding profile was similar between the two groups. However, binding to the p27 containing peptide (F 101–121) and peptide F 425–450 was higher with sera from infected infants with RSV-neutralizing antibodies.

Adolescent sera exhibited expanded binding profile to more F derived peptides. All samples (100%) bound to the F-2.1 p27 antigenic site (aa 101–121) (mean RU = 105), and also to F1.2 peptide (aa 23–74) that includes part of site ϕ (mean RU = 42) and to C-terminus F-5 peptide (aa 425–450) that spans previously defined antigenic site IV (mean RU = 45). In addition, 67% of sera reacted with F-3.1 peptide (aa 176–240), which contains a major part of site ϕ, a site that was also recognized by pre-fusion site ϕ specific MAb D25 (Fig 1). Binding to other F peptides varied, ranging between 0%-25% of the positive samples (Fig 5B and S2 Table).

Adult sera showed further expansion of the antibody binding repertoire against the F peptides (Fig 5C). Again, 100% of samples reacted with F-2.1 p27 site (aa 101–121) but with lower antibody levels (mean RU = 45) compared with the younger age groups. The majority of sera bound to several C-terminus antigenic sites; F-4.1 (aa 310–368) and F-6.1 (aa 471–493) (mean RU = 35 and 15, respectively). Binding to F-5 antigenic site peptide (containing the previously described site IV) was measured in 67% of samples, and binding of peptides that include site ϕ (aa 23–74 and aa 176–240) was seen with 92% and 33% of the adult samples, respectively. There was also an increase in binding antibodies to peptide F-3.2 (aa 234–287), which contains antigenic site II (Palivizumab binding sequence), among adult sera. Antibody reactivity to the F-2.1 p27 peptide was significantly lower in adults compared with children and adolescents (Fig 5C).

As shown in Fig 4, analysis of post-infection sera using GFPDL containing RSV-G gene sequences primarily identified large regions flanking the CCD motif. However, peptides were synthesized only for antigenic sites with maximum length of 50 amino acids. As shown in Fig 5D and 5F, binding to G-derived peptides was more restricted in all age groups using SPR analysis compared with the GFPDL analysis. All sera from the <2 yr aged children with confirmed RSV infection (PRNT titers >40) showed strong reactivity with a G peptide in antigenic site G-4 that includes the CCD motif (aa 169–207) (mean RU = 334) (Fig 5D and S2 Table). Infants <2 years with no evidence of RSV infection (absence of RSV neutralization antibodies) showed positive but lower reactivity to peptide G 169–207 compared with sera from seropositive children (S3D and S3C Fig respectively).

RSV-G specific antibodies in adolescent sera were again focused in and around the CCD region, with 100% of samples reacting with G-4 peptide (aa 169–207) (mean RU = 394) and also with the preceding N-terminal antigenic site G-3.1 peptide (aa 148–178) (mean RU = 52) (Fig 5E and S2 Table). Interestingly, while adult sera reacted with the same antigenic site peptides as the younger groups (aa 169–207 and aa 148–178), the total binding to immunodominant G-4 site was significantly lower (mean RU = 154) compared with either the <2yr children (*p = 0*.*0009*) or the adolescent group (*p = 0*.*0001*) (Fig 5F and S2 Table). Together, these SPR analyses demonstrated expansion of the anti-F antibody repertoire and contraction of the anti-G antibody response in adults compared with young children and adolescents.

### SPR analysis of sera binding to pre-fusion vs. post-fusion form of F protein and to non-glycosylated vs. glycosylated G proteins

The series of antigenic site peptides based on the GFPDL analyses were tested using SPR. However, not all the large conformational antigenic sites identified using GFPDL panning could be individually synthesized and analyzed. Therefore, we also measured the binding of individual serum samples from the same three age cohorts to different forms of properly folded RSV-F and G proteins. The pre-fusion F protein (DS-Cav1) [27, 33] and post-fusion F proteins were expressed in mammalian cells. The G protein was expressed either in mammalian cells (glycosylated) or in bacterial system (unglycosylated) as previously described [30]. In preliminary study, we confirmed that these proteins are properly folded and can be recognized by anti-F and anti-G specific MAbs. As shown in S4 Fig, MAb 131-2G bound equally to both glycosylated and unglycosylated G proteins but not to any of the F proteins (panel A). Pre-fusion specific MAb D25 (site ϕ) bound only to pre-fusion F but not to post-fusion form of F or to the G proteins (S4B Fig), while MAb Palivizumab (site II) bound to both pre-fusion and post-fusion F proteins but not to the RSV-G proteins (S4C Fig). The specificity of MAbs binding was important to demonstrate that all four recombinant proteins displayed native epitopes. In addition, the purified G proteins formed tetramers[30].

**Fig 6:**
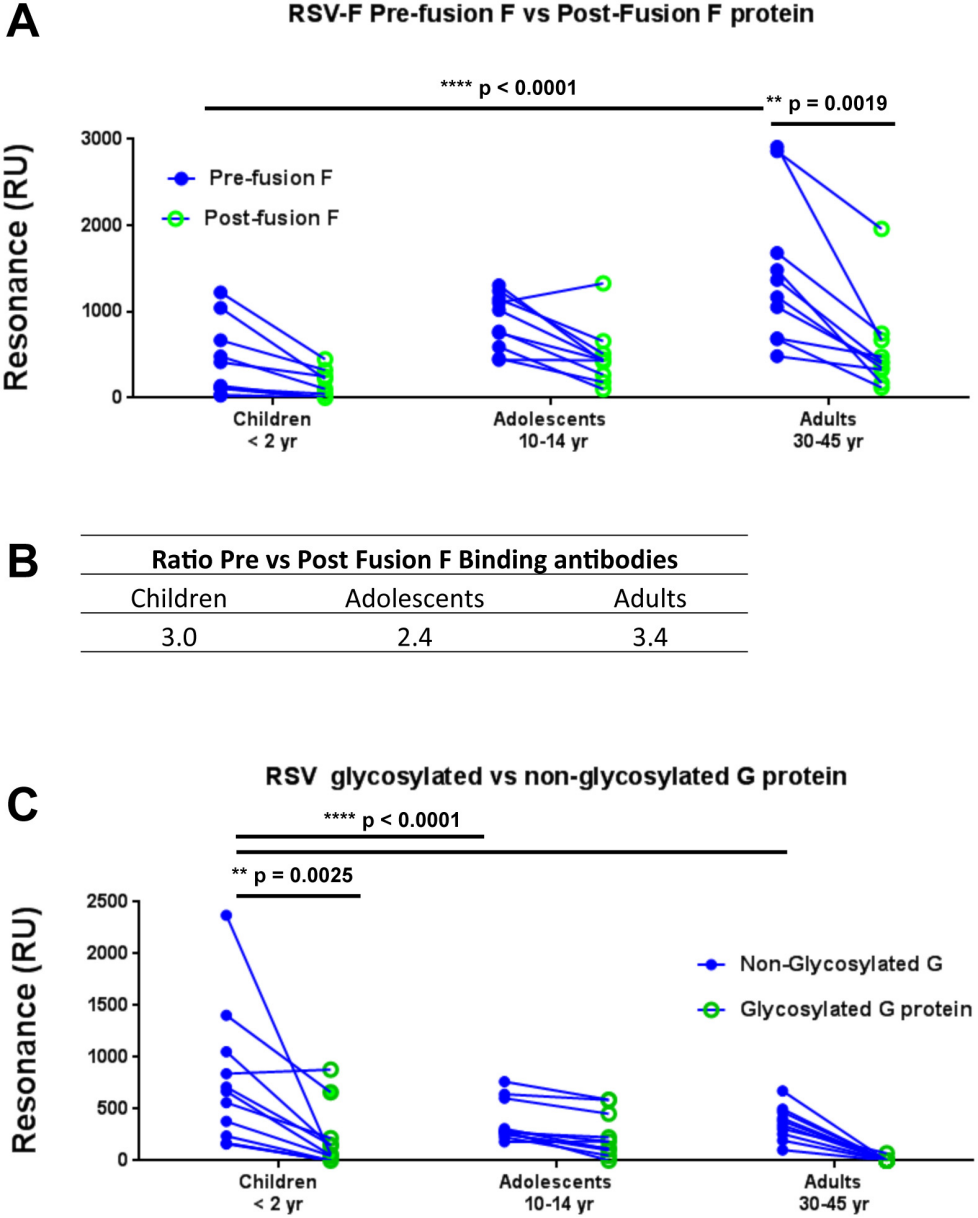
SPR based analysis of RSV-F and RSV-G purified proteins reactivity with human sera from of different age groups. (A) Serum samples collected from children (< 2 years), adolescents (10–14 years), and adults (30–45 years), were analyzed for total binding to purified pre-fusion RSV-F (blue) or post-fusion RSV-F (green) protein by SPR. The pre-fusion RSV F (DS-Cav1) was produced in 293F cells and encodes amino acids 26–513Δ110–136. Post-fusion RSV-F protein was obtained from Sino biologicals and encodes amino acids 22–529Δ110–136. (B) Ratio of serum binding to RSV pre-fusion vs post-fusion F protein in different age groups by SPR. (C) Binding to non-glycosylated RSV-G (blue) vs glycosylated RSV-G (green). The RSV-A2-G (67–298) ectodomains were produced in bacterial or mammalian 293F cells. Total antibody binding is represented in SPR resonance units. Individual sample reactivity to different F proteins (A) or to RSV-G proteins (C) are shown by connected lines. Statistical significant differences of antibody reactivity between different age groups or within group are depicted with *p* values.

It was recently suggested that site ϕ, which is only expressed on the pre-fusion form of the F protein [27, 33, 34], is a dominant target of neutralizing antibodies in post-infection human sera, and pre-fusion F likely represents the native virion-associated fusion protein that is responsible for mediating RSV infection[35]. Therefore, we compared the binding of individual serum samples in the three age groups with the pre-fusion vs. post-fusion recombinant F protein in SPR-based real time kinetic analysis (Fig 6A and 6B). For most samples, serum antibody binding to the pre-fusion F protein was significantly higher than to the post-fusion F protein (blue vs. green circles) in all the age groups (*p = 0*.*0001* for children & adolescent, and *p = 0*.*0019* for adults) (Fig 6A). The ratios of pre-fusion to post-fusion F antibody binding (Max RU) averaged 3.0 for the <2yr, 2.4 for the adolescent group, and 3.4 for the adult group (Fig 6B). In addition, we found a significant increase in the antibody binding levels to the pre-fusion F in the adult group (30–45 yr) compared with the young children (<2yr) and adolescents in agreement with expansion of the antibody repertoire observed in the SPR analyses of antigenic site peptides (Fig 5A–5C).

We also evaluated binding of individual serum samples to RSV-G protein. The native G protein is highly N- and O-linked glycosylated. The filamentous phage display libraries are produced in a bacterial system and some of the phage-expressed segments may be masked by glycans in the native G proteins. Therefore, we compared antibody binding of individual sera with the properly folded recombinant non-glycosylated G (produced in a bacterial system) with binding to the fully glycosylated G protein produced in a mammalian system [36] (Fig 6C). In all age groups, binding to non-glycosylated G was higher than to the glycosylated counterpart. Most importantly, the highest anti-G antibody binding levels were observed in the <2yr children. Anti-G antibody binding declined significantly in the adolescent and adult groups similar to the decline in serum antibody reactivity against the individual G antigenic site peptides in Fig 5. The unglycosylated G was previously shown to generate protective immunity against homologous and heterologous RSV strains in BALB/c mice. Importantly, the antibodies elicited by the unglycosylated G bound well to glycosylated G [30]. Therefore, measurements of anti-G antibody binding using both forms of the G protein allowed us to capture the full array of functional antibodies that were elicited by natural RSV infections.

Together, our study demonstrated independent evolution of antibody binding patterns to RSV F and G proteins following primary/early viral infection in the very young (<2yr) and in adolescent and adult age groups.

## Discussion

The development of an effective RSV vaccine is of prime importance. The primary target populations include infants < 6 months of age and children under 2 years who are most likely to suffer from severe RSV disease and complications [5]. In addition, maternal vaccination to induce sufficiently high titers of protective antibodies that can cross the placenta could be a safe and effective strategy to delay infection in the newborn and reduce complications from primary RSV infection [20]. Also, polyclonal and monoclonal antibodies targeting protective epitopes in the F and G proteins are under clinical development [27, 34, 37, 38] [39, 40]. Therefore, it is important to obtain an in-depth understanding of the pre-existing antibodies in infants and their immune responses following primary RSV infection in order to identify antibody responses that may best correlate with protection and facilitate the development of effective countermeasures against RSV disease.

To that end, we used whole-genome-phage-display libraries (GFPDL) to unravel the antibody epitope repertoires of polyclonal serum antibodies from infants with no evidence of RSV infection, and to follow the development of the antibody profiles after primary or early RSV exposure. The F- and G-GFPDLs provided an unbiased comprehensive approach to identify the spectrum of both linear and conformational epitopes on the surface of the RSV membrane proteins that are recognized by the antibodies elicited by primary RSV infection. We initially tested a panel of 16 MAbs against RSV F and G that recognized both linear and conformational epitopes and mapped the epitopes of all of these MAbs using our GFPDLs (data shown for representative MAbs in Fig 1).

As expected, pre-infection sera from young children (≤ 9 months) contained IgG antibodies against both F and G that represented residual maternal antibodies as previously reported [14, 15, 32]. Following RSV infection these children developed neutralizing antibody titers and had anti-RSV IgA responses indicative of newly acquired immunity. Interestingly, affinity selection with F-GFPDL using pooled pre-infection vs. post-infection infant sera resulted in similar numbers of captured phages. However, we found some changes in the epitope profiles with increase in frequency of inserts mapping to the N-terminal region including part of site ϕ (expressed on pre-fusion F), to p27 (found only in unprocessed F0), and to the C-terminus of F bound by the post-RSV primary/early infection children sera. Binding to site F-3.2 (targeted by Palivizumab) was seen with both pre-and post-RSV infection polyclonal antibodies.

In contrast to the modest increase in the number of F binding phage clones, panning with G-GFPDL with post-infection sera resulted in 100-fold increase in total bound phages all across the G regions. The most pronounced increases were seen in the frequency of clones mapping to the conserved CCD region containing antigenic site G-4 (aa 169–207) and to a region immediately amino-terminal to the CCD defined by antigenic site G-3. We hypothesize those IgG maternal antibodies that pass the placenta may last up to 9 months in the newborn, could interfere with the primary immune response to first infection (or vaccination). If the maternal antibodies are more biased towards reactivity to the F protein compared with G protein on the virions, a stronger anti-G naïve response could ensue during primary infections.

Based on the GFPDL results, we synthesized large set of peptides representing most of the key antigenic sites in F and G that were recognized by the pooled post-infection sera. These peptides were used in real-time SPR to measure binding of panels of serum from young children (<2 yr), adolescent (10–14 yr), and young adults (30–45 yr). The strongest binding in the youngest group was to the p27 peptide (aa 101–121) in F-2.1, with significantly lower reactivity against other F peptides including sites F-3.2 (site II) and F-5 (site IV). The epitope-binding patterns in the F antigenic sites were broader for the adolescent and adult samples, suggesting expanding B cell repertoires, which could result from repeated RSV exposures. The strong binding to the novel F-2.1 p27 epitope was not previously reported, albeit Burton *et al* reported a strong antibody response to uncleaved RSV-F protein in post-infection sera [41]. Since p27 is excised during processing of F0 into F1/F2, it is not part of the mature F protein on virions. Therefore, the strong anti-p27 response most likely resulted from exposure of the immune system to immature virions and/or dying virus-infected cells during the course of a productive infection. This observed high anti-p27 antibody response suggests an immune pressure mediated high mutation rate observed in the p27 sequence of circulating RSV strains [42]. In the very young, anti-p27 reactivity may be a biomarker of a recent RSV infection and could be used for development of RSV serodiagnostic assay. By using GFPDL analysis, we also identified two novel epitopes in antigenic sites 2 and 3 that are specific for pre-fusion F compared with post-fusion F protein and were not previously identified.

One of the possible limitations of GFPDL-based assessments is that while the phage display is likely to detect both conformational and linear epitopes on RSV-F and G, they are unlikely to detect paratopic interactions that require post-translational modifications and rare quaternary epitopes that cross protomers (e.g., those created by trimers of F or tetramers of G expressed on virus surface). However, in the preliminary study with polyclonal antibodies from vaccinated rabbits 86–89% of anti-F and anti-G antibodies were removed by adsorption with the corresponding GPDLs, supporting the use of the F-GFPDL and G-GFPDL for analyses of human sera. Moreover, binding to properly folded F and G proteins in real time kinetics based SPR can overcome these limitations and provide additional insight into the post-infection anti-RSV polyclonal antibody response.

The commercial RSV IgG RespiGam (Medimmune) made from the sera of human subjects with high RSV-neutralizing titers was shown to include antibodies targeting the pre-fusion F, and these antibodies contained most of the anti-RSV neutralizing activity *in vitro* [43]. We found that antibodies against both pre-fusion and post-fusion F were higher in the adults compared with the younger age groups. Importantly, irrespective of the age group, the binding of individual serum samples to pre-fusion F protein was 2–3 folds higher than against the post-fusion F protein. These data confirmed that RSV infections induce stronger responses against the pre-fusion F protein with gradual increase in anti-F antibody levels with age and a general expansion of the antibody repertoires probably driven by repeat RSV exposures.

In contrast to the broadening anti-F response over age, anti-G antibody binding in all age groups was primarily focused on the antigenic sites G-4, encompassing the CCD motif (aa 169–207), and to the preceding/overlapping antigenic site G-3 (aa 148–178) in the SPR assay. However, there are a few caveats to this finding. The GFPDL analysis identified several large immunodominant antigenic sites flanking the CCD motif (G-2 and G-5 sites in Fig 4) that could not be chemically synthesized or individually expressed. Therefore, most of the anti-G binding activity observed using G-GFPDL was lost when shorter synthetic linear peptides were used in the SPR binding assay (Figs 4 vs. 5). Previously study also found highly restricted binding to short peptides (15mers) spanning the C-terminus of G protein [17].

Another observation made in the current study was a significant decline in the levels of antibody binding to antigenic site G-4 (containing the CCD) in adults compared with young children and adolescents (Fig 5). To confirm this finding, we also measured the binding to intact G proteins that were either unglycosylated (expressed in a bacterial system) or fully glycosylated (expressed using mammalian cells)[30]. Binding to unglycosylated G was higher than to the glycosylated G, in agreement with a previous study [19]. Importantly, we found significant reduction in anti-G protein binding antibodies with adult samples compared with the younger groups similar to observation with the G-4 peptide (169–207). Lower antibody levels against G in the adult group cannot be simply explained by exposure to RSV with G variants since the CCD domain is highly conserved among all RSV strains. It is also the least glycosylated region of the G protein.

Long term maintenance of humoral immunity is provided by a combination of long lived antibody plasma cells (LLPC) and memory B cells (MBC) [44–46].[47, 48]. [48][16]). Falsey *et al*. described serum antibody decay against RSV in adults following natural RSV infections [24]. However, their study did not compare antibody decay against F vs. G proteins. In our study, we identified significantly lower anti-G binding levels in adults, including antibodies targeting the conserved CCD domain, suggesting a differential decay of anti-G LLPC or MBC compared with anti-F LLPC/MBC following RSV infections. This finding was not previously described and should be taken into consideration for development of vaccines targeting older individuals.[30, 49, 50]

**Fig 7:**
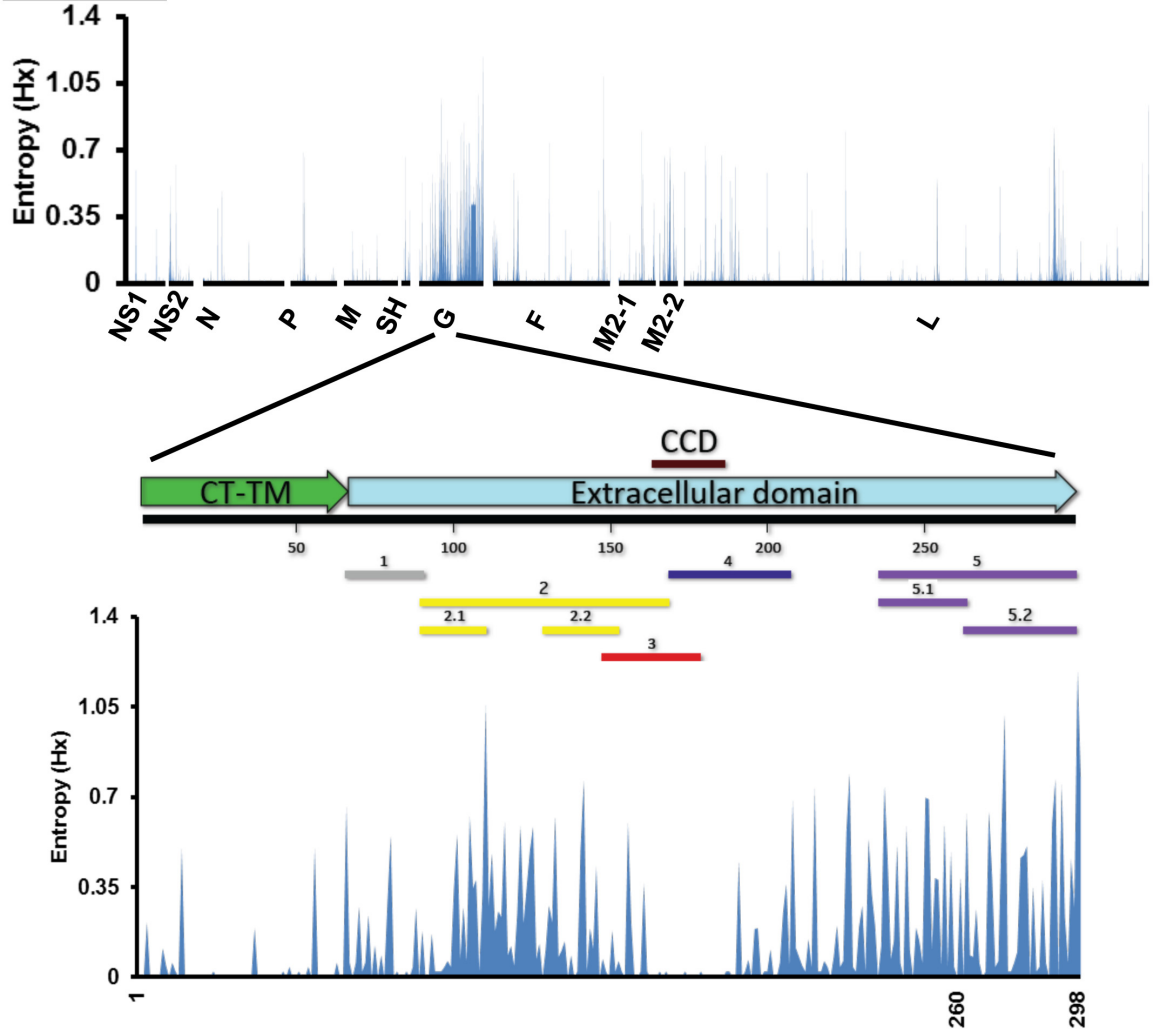
Contribution of anti-G antibody response following RSV primary infection in the evolution of RSV viruses. Accumulation of amino acid variations in RSV protein sequence in human isolates collected from 1979–2013. Protein sequences from RSV subtype A human isolates were obtained through the Virus Pathogen Resource (ViPR, http://www.viprbrc.org). 297 isolates with complete genome sequences and non-ambiguous amino acids were included in the analysis. The sequences were aligned using MUSCLE and a consensus sequence was created based on the alignment. The polymorphisms from that consensus were then scored by calculating the entropy (Hx) using Bioedit. Entropy scores are represented in the Y-axis and the RSV proteins in the X-axis. Alignment of antigenic sites in RSV-G identified using GFPDL approach with sequence variability scores in the RSV-G protein is shown the bottom half.

Evolution of RSV demonstrated preferential diversification of the G gene with a mutation rate of 5% compared with other genes within the RSV viral genome (Fig 7). Molecular evolution studies on RSV-G gene in circulating viral strains has shown accumulation of mutations with non-synonymous changes mainly in the N-terminus and C-terminus regions flanking the CCD motif but not in the central conserved region, which is functionally required for virus attachment to the cell (Fig 7) [9, 10, 51, 52]. It was previously suggested that the immune system contributes to the progressive accumulation of amino acid changes in the N- and C-terminus of the G protein ectodomain, but non-immune mechanisms were also postulated [9, 51–53]. Our results with the G-GFPDL following early RSV infection demonstrated significant antibody binding to the G antigenic regions (G-1, G-2 and G-5) undergoing diversification (Fig 4). These findings provide evidence in support of the theory that immune pressure plays an important role in selection of non-synonymous changes in the G gene that apparently do not compromise its function as a key attachment protein.

Together, our study suggests an unlinked evolution of the antibody responses to F and G proteins in humans. The significant drop in anti-G antibody levels in adults may be a factor in sustained susceptibility to RSV infections throughout life. Live attenuated RSV vaccines under development are expected to elicit balanced anti-F and anti-G antibodies [49]. On the other hand, several current subunit/particle based vaccines under development contains F protein only [54, 55]. The implications of our findings suggest the need to include G proteins in future RSV vaccines in order to boost the anti-G responses. We have previously demonstrated that unglycosylated G protein produced from bacterial system elicited stronger protective immunity against homologous (RSV A2) and heterologous (RSV B) challenge compared with the glycosylated G protein produced in mammalian cells [30]. G proteins from currently circulating strains could enhance the protective immune responses against future RSV strains.

## Methods

### Sera and monoclonal antibodies

Monoclonal antibody (MAb) Palivizumab (anti RSV-F) was purchased from the NIH Pharmacy, MAb D25 (anti RSV-F) was a gift from Peter Kwong (VRC, NIH), and MAb 131-2G (Anti RSV-G) was purchased from Millipore. Human serum samples were previously obtained from subjects 30–45 years old (adults) (N = 12), 10 to 14 years (adolescents) (N = 12), and 6 to 18 months (children) (N = 30) of age following parental informed consent. Samples are anonymous, and permission to test these de-identified samples for RSV neutralizing antibody was obtained from the U.S. Food and Drug Administration’s Research Involving Human Subjects Committee (FDA-RIHSC) under exemption protocol #12-079B.

### LIPS assay to detect RSV-N specific IgG antibodies

COS-1 cell lysates containing Ruc-tagged RSV-N protein (Ruc-N) or Ruc only (without insert) were generated and LIPS assay performed as previously described [31]. A positive response (RLU of 8000 or above) was defined as 5 standard deviations above the mean value obtained with control sera tested against Ruc-N antigen. Antisera were tested in at least two independent assays and in parallel against Ruc antigen without insert to confirm that reactivity was specific for RSV-N antigen.

### RSV-Plaque Reduction Neutralization Test (RSV-PRNT)

RSV-PRNT was performed as previously described [36, 56]. Briefly, serial 4-fold dilutions of heat-inactivated serum were mixed with virus (diluted to yield 20–50 plaques/well) containing 10% guinea pig complement (Rockland Immunochemicals; Philadelphia, PA) and incubated for 1 hr at 37°C. Antibody-virus mixtures were inoculated in duplicate onto HEp-2 cell monolayers. The 50% endpoint titers in the PRNT assay were determined using the Spearman-Kärber method[56].

### Rabbit vaccination

Female New Zealand rabbits were vaccinated three times (28 days apart) with recombinant F (pre fusion) or G (unglycosylated)[30] proteins from RSV A2 strain (50 μg/dose) adjuvanted with Emulsigen. Post third vaccination sera were used for GFPDL analysis as well as for adsorption of F-GFPDL and G-GFPDL. Pre- and post-adsorption sera were evaluated by SPR for binding to the F and G proteins.

### Construction of RSV whole genome-fragmented phage display libraries (GFPDL)

cDNA corresponding to RSV-F and RSV-G gene segments of the A2 subtype strain were chemically synthesized and used for cloning. fSK-9-3 is a gIIIp display based phage vector where the desired polypeptide can be expressed as gIIIp fusion protein.

Phage display libraries were constructed individually for RSV-F and RSV-G genes. Purified DNA containing RSV-F and RSV-G were digested with DNase, and used for GFPDL construction. Since the phage libraries were constructed from the whole RSV-F and G surface glycoprotein genes, they potentially display all possible viral protein segments ranging in size from 15 to 250 amino acids, as fusion protein on the surface of bacteriophage.

### Affinity selection of RSV F- and G-GFPDL phages with RSV pre- and post-exposure polyclonal children sera

Prior to panning of GFPDL with polyclonal serum antibodies, serum components that could non-specifically interact with phage proteins were removed by incubation with UV-killed M13K07 phage-coated Petri dishes.

For RSV-F and RSV-G GFPDL panning using pre-RSV infection human sera, equal volumes of serum from children 9 months of age with PRNT titers <40 and with LIPS-N values <8000 RLU were pooled. For GFPDL panning of post-RSV exposure, equal volumes of human sera from children 15/18 months of age with PRNT titers ≥ 40 and LIPS-N values >8000 RLU were pooled. Subsequent GFPDL affinity selection was carried out in-solution (with Protein A/G) as previously described [26]. DNA of 768 phage clones were sequenced for each sera with individual GFPDL.

### RSV-F proteins

The pre-fusion RSV F (DS-Cav1) plasmid encoding amino acid residues 26–513Δ110–136 of RSV-A2 strain was a kind gift from Peter Kwong (VRC, NIH). DS-Cav1 protein was produced in 293F cells[27] Post-fusion RSV-F protein encodes amino acid residues 22–529Δ110–136 of RSV-A2 strain fused to a polyhistidine tag produced in insect cells with endotoxin levels <1 EU/μg of protein and was obtained from Sino Biologicals.

### Production of recombinant glycosylated G protein using 293 Flp-In cells

RSV-G gene codon optimized for mammalian cells was chemically synthesized. *Not*I and *Pac*I sites were used for cloning the RSV A2 G ectodomain coding sequence (67–298) in the pSecR vector for mammalian expression to express G protein in 293Flp-In cells. 293-Flp-In cells and pOG44 (plasmid expressing Flp-In recombinase) were obtained from Invitrogen (Carlsbad, CA). The 293-Flp-In cell line stably expressing the RSV A2 G protein with secretory signal peptide from IgG kappa chain was developed as described previously [57]. Briefly, 293-Flp-in cells were co-transfected with the plasmids expressing Flp-in recombinase and the RSV A2 G ectodomain in DMEM medium (Invitrogen). Twenty four hours after transfection, culture medium was replaced with fresh DMEM containing 150 μg/mL of hygromycin for selection of stably transfected cells. For protein expression, cells were maintained in 293-Expression medium (Invitrogen), and culture supernatant was collected every 3–4 days. Supernatant was cleared by centrifugation and filtered through a 0.45 μm filter before purification through a His-Trap FF column (GE healthcare).

### Production of recombinant unglycosylated G protein from *E*. *coli*

Recombinant RSV G 67–298 extracellular domain was expressed in *E*. *coli* BL21(DE3) cells (REG) and was purified under controlled redox refolding conditions [30]. Endotoxin levels of the purified protein were <1 EU/μg of protein. Both proteins were previously described [30].

### Surface Plasmon Resonance (SPR)

Steady-state equilibrium binding of polyclonal antibodies in human sera was monitored at 25°C using a ProteOn surface plasmon resonance biosensor (BioRad). The purified recombinant F or G proteins were coupled to a GLC sensor chip via amine coupling with 200 resonance units (RU) in the test flow channels and spatial density of the proteins were optimized such that there is single monovalent interaction for each antibody molecule irrespective of their isotype. For peptide analysis in SPR, chemically synthesized biotinylated RSV-F or RSV-G peptides were captured on NLC chips. Samples of 300 μl freshly prepared sera at 10-fold dilution or MAbs (starting at 1 μg/ml) were injected at a flow rate of 50 μl/min (120 sec contact duration) for association, and disassociation was performed over a 600-second interval. Responses from the protein surface were corrected for the response from a mock surface and for responses from a buffer-only injection. Total antibody binding and data analysis results were calculated with BioRad ProteOn manager software (version 2.0.1).

### Statistical analyses

The statistical significances of group differences were determined using a one-way ANOVA and Bonferroni multiple comparisons test. Correlations were calculated with a Spearman’s two-tailed test. p-values less than 0.05 were considered significant with a 95% confidence interval.

### Ethics statement

The study at CBER, FDA was conducted with de-identified samples under Research Involving Human Subjects (RIHSC) exemption 12-079B; and all assays performed fell within the permissible usages in the original consent.

## Supporting Information

S1 Table Seroconversion of paired infant sera samples after primary RSV infection. (TIF)

S2 Table Antigenic sites in RSV F and G protein. (TIF)

S1 Fig GFPDL analyses of Rabbit pre- and post-vaccination sera. New Zealand rabbits were vaccinated three times (28 days apart) with recombinant F (prefusion) or G (unglycosylated)[30] proteins from RSV A2 strain (50 μg/dose) adjuvanted with Emulsigen. Pre-vaccination and immune sera post third vaccination were used for GFPDL analysis (A) total number of F-GFPDL phages captured by pre-vaccination vs. post vaccination sera (B) Distribution of phage clones after RSV-F GFPDL affinity selection with sera obtained pre-vaccination and post third vaccination with recombinant post-fusion F protein. The amino acid designation is based on the RSV-F protein sequence. Bar location indicates the homology of the displayed RSV-F protein sequence on the phage clones after affinity selection. The thickness of each bar represents the frequencies of repetitively isolated phage inserts (only clones with a frequency of two or more are shown) (C) Distribution of phage clones after RSV-G GFPDL affinity selection with sera obtained pre-vaccination and post third vaccination with recombinant G protein
(TIF)

S2 Fig Isotype of total antibody binding to RSV F and G proteins in Infants following RSV primary infection. The isotype of serum antibodies bound to RSV pre-fusion form of F protein (DS-Cav1) in (A) or RSV-G protein (B) are shown for the serum from children following RSV primary infection as measured in SPR experiment.
(TIF)

S3 Fig Surface plasmon resonance (SPR) analysis of human sera from of young children (< 2 year) with prior RSV infection (determined by positive PRNT neutralization assay) vs. uninfected (no neutralizing titers) for binding to peptides from different antigenic sites within RSV-F and RSV-G. Selected peptides of RSV-F and G proteins representing the antigenic sites (same as in Fig 5) were chemically synthesized and tested for binding against individual sera samples using real time SPR kinetics experiment. Total antibody binding is represented as SPR resonance units (RU). Panels A-B show total antibody binding against the F peptides and panels C-D show total antibody binding to G peptides with prior RSV infection (determined by positive PRNT neutralization assay) in panels A & C vs. uninfected (no neutralizing titers) in panels B and D.
(TIF)

S4 Fig Specific binding of anti-F and anti-G MAbs to recombinant F and G proteins. MAbs 131-2G (anti-G) (A), D25 (anti-F pre-fusion form only) (B), and Palivizumab (anti-F site II reactive with pre-fusion and post-fusion forms) (C) were analyzed for total binding to purified pre-fusion RSV-F (red), post-fusion RSV-F (blue), non-glycosylated RSV-G (black), and glycosylated RSV-G (green) proteins. Total antibody binding is represented in SPR resonance units.
(TIF)
