# Supplementary figures and images for: Antigenic Fingerprinting following Primary RSV Infection in Young Children Identifies Novel Antigenic Sites and Reveals Unlinked Evolution of Human Antibody Repertoires to Fusion and Attachment Glycoproteins
Source: PLoS Pathog. 2016 Apr 21;12(4):e1005554. doi: 10.1371/journal.ppat.1005554 (PMC4839671; doi:10.1371/journal.ppat.1005554)

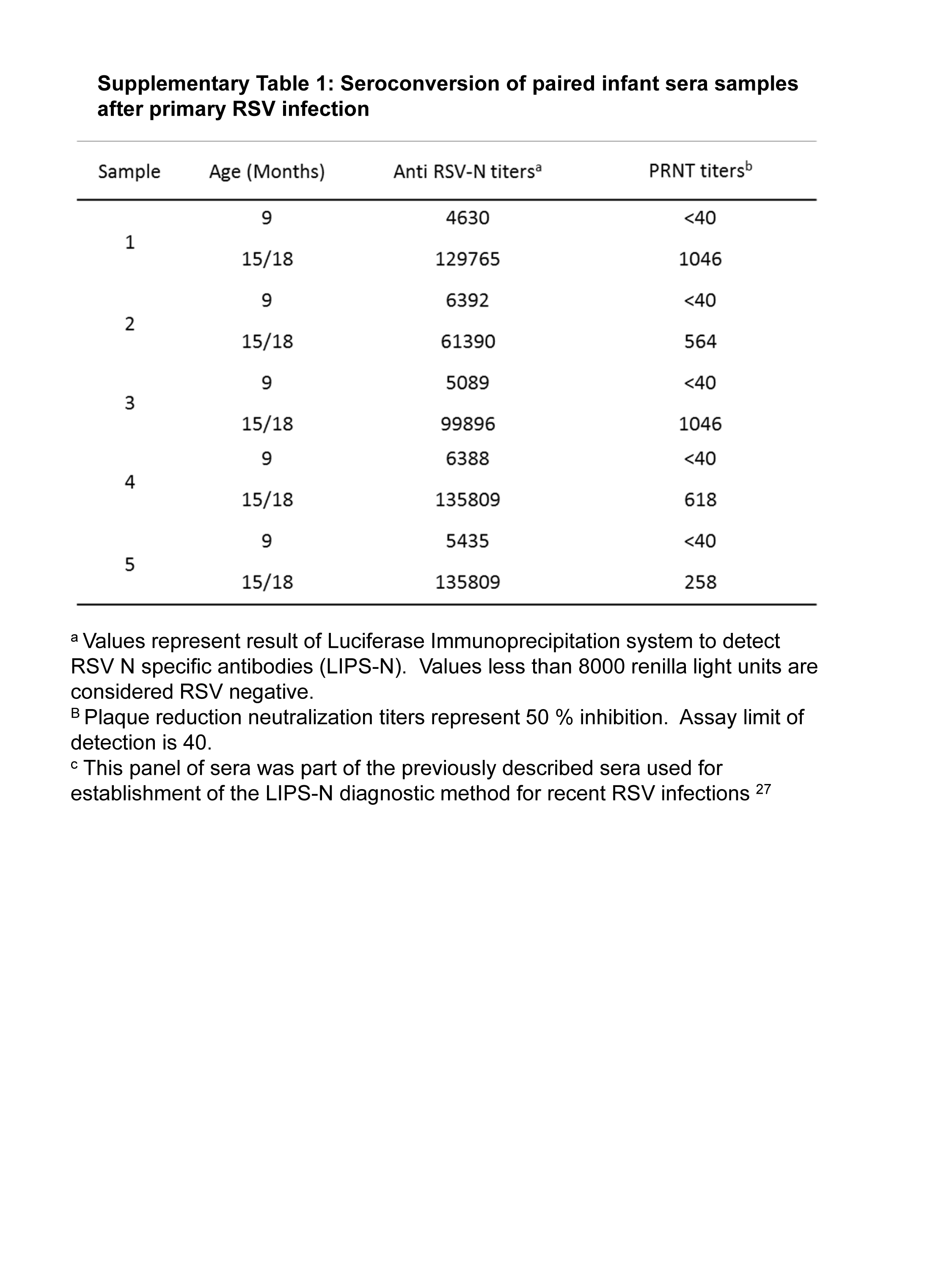

Supplement: S1 Table — (TIF) [file ppat.1005554.s001.tif]

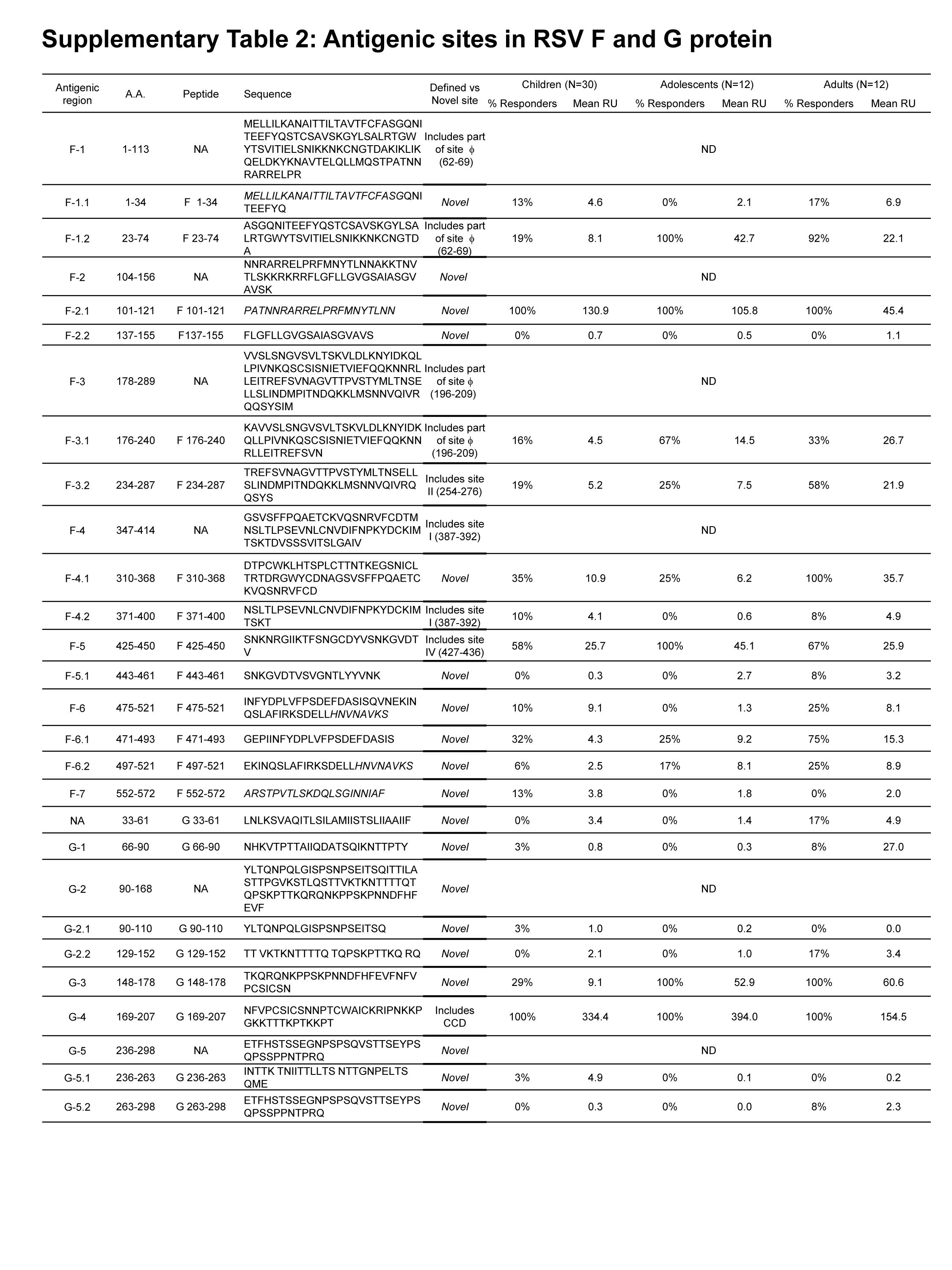

Supplement: S2 Table — (TIF) [file ppat.1005554.s002.tif]

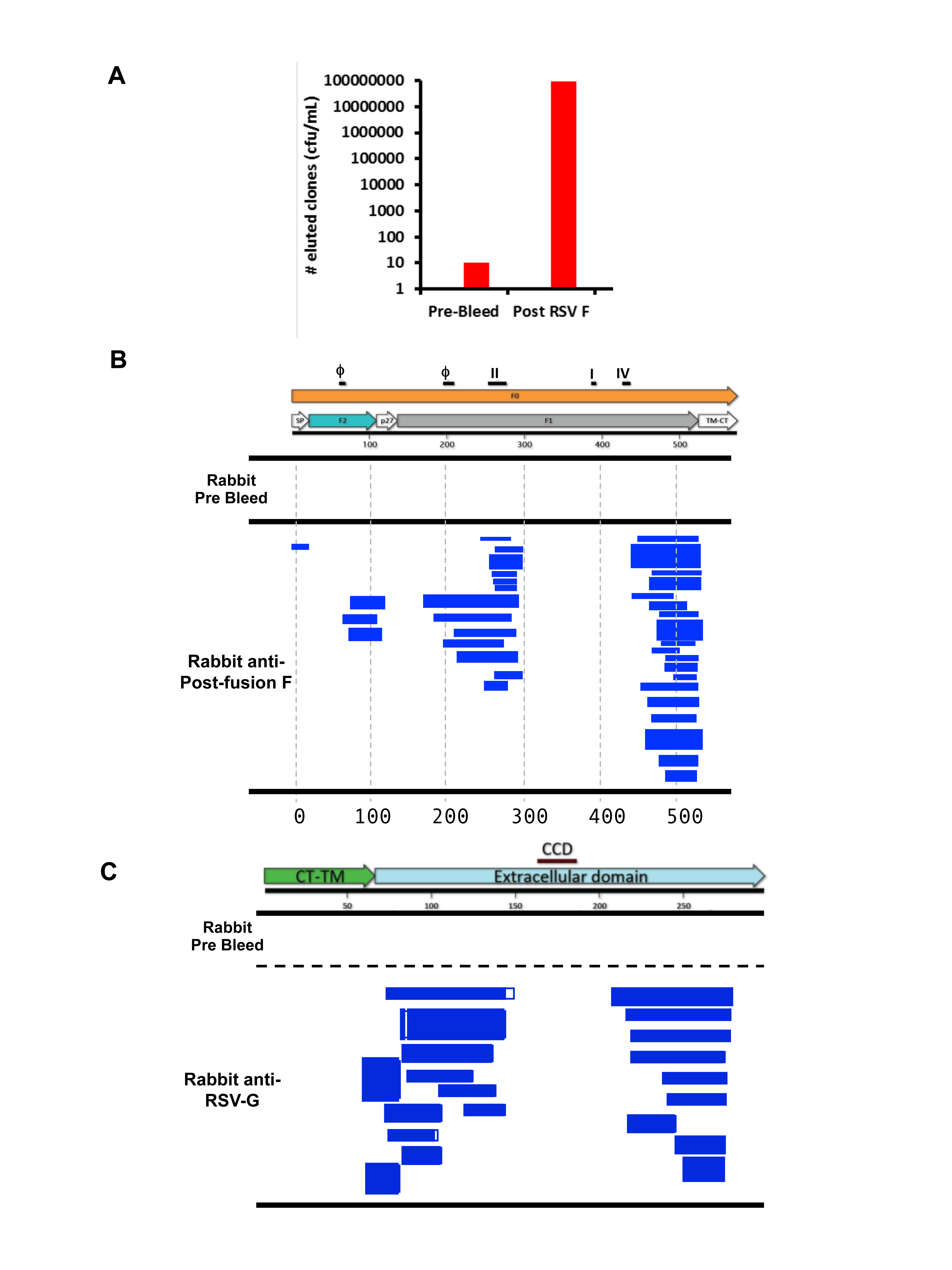

Supplement: S1 Fig — New Zealand rabbits were vaccinated three times (28 days apart) with recombinant F (prefusion) or G (unglycosylated)[30] proteins from RSV A2 strain (50 μg/dose) adjuvanted with Emulsigen. Pre-vaccination and immune sera post third vaccination were used for GFPDL analysis (A) total number of F-GFPDL phages captured by pre-vaccination vs. post vaccination sera (B) Distribution of phage clones after RSV-F GFPDL affinity selection with sera obtained pre-vaccination and post third vaccination with recombinant post-fusion F protein. The amino acid designation is based on the RSV-F protein sequence. Bar location indicates the homology of the displayed RSV-F protein sequence on the phage clones after affinity selection. The thickness of each bar represents the frequencies of repetitively isolated phage inserts (only clones with a frequency of two or more are shown) (C) Distribution of phage clones after RSV-G GFPDL affinity selection with sera obtained pre-vaccination and post third vaccination with recombinant G protein (TIF) [file ppat.1005554.s003.tif]

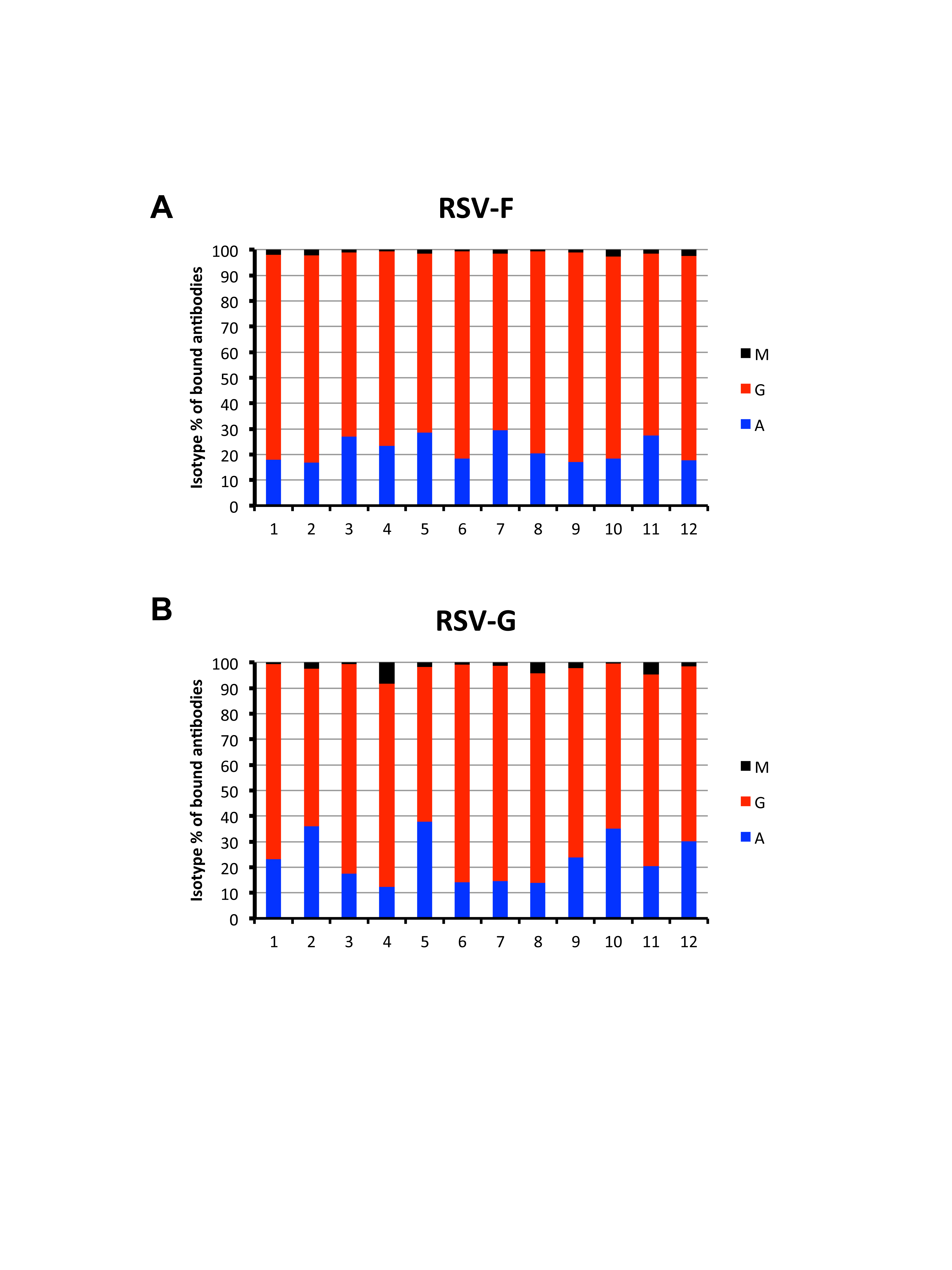

Supplement: S2 Fig — The isotype of serum antibodies bound to RSV pre-fusion form of F protein (DS-Cav1) in (A) or RSV-G protein (B) are shown for the serum from children following RSV primary infection as measured in SPR experiment. (TIF) [file ppat.1005554.s004.tif]

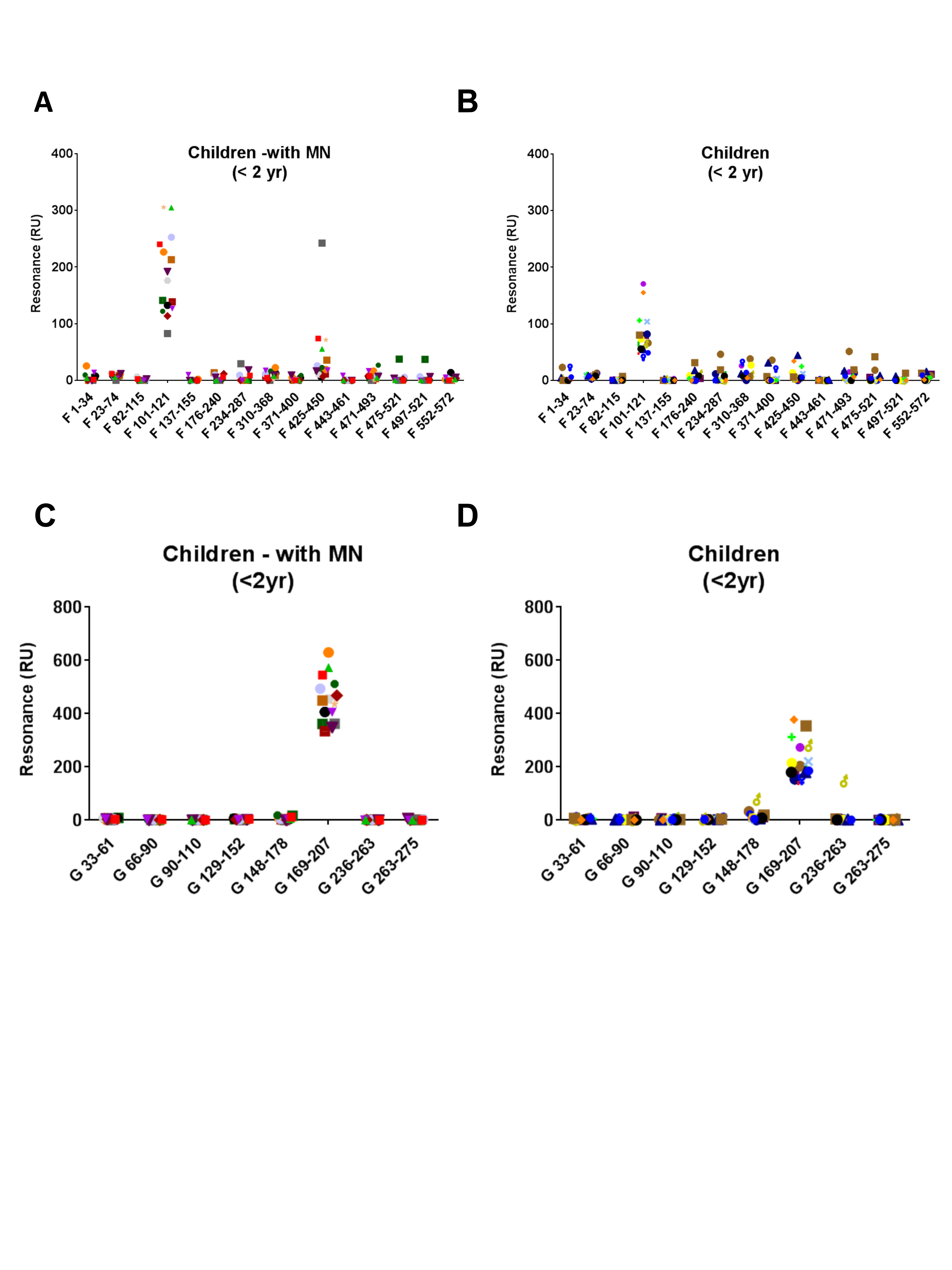

Supplement: S3 Fig — Selected peptides of RSV-F and G proteins representing the antigenic sites (same as in Fig 5) were chemically synthesized and tested for binding against individual sera samples using real time SPR kinetics experiment. Total antibody binding is represented as SPR resonance units (RU). Panels A-B show total antibody binding against the F peptides and panels C-D show total antibody binding to G peptides with prior RSV infection (determined by positive PRNT neutralization assay) in panels A & C vs. uninfected (no neutralizing titers) in panels B and D. (TIF) [file ppat.1005554.s005.tif]

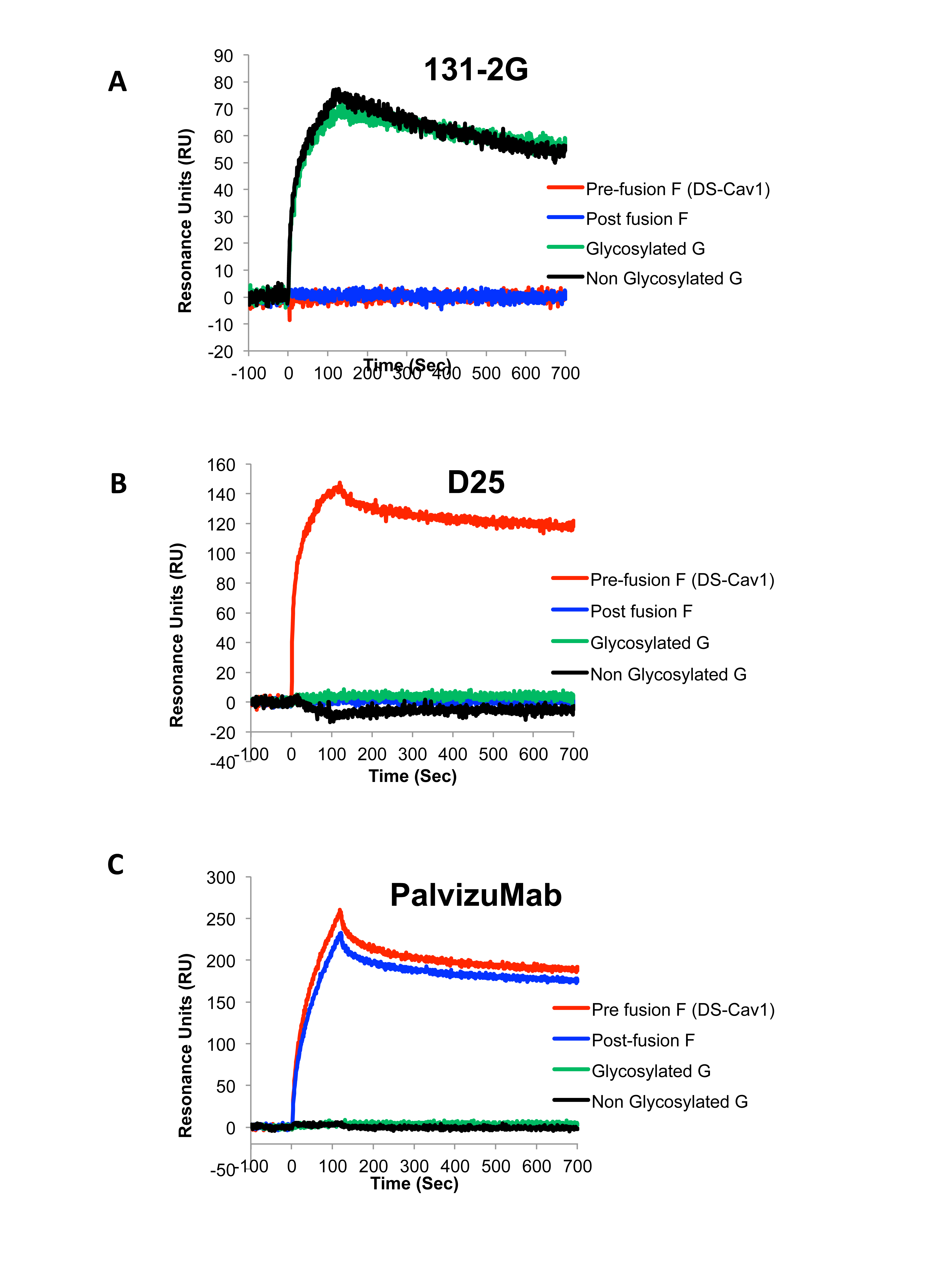

Supplement: S4 Fig — MAbs 131-2G (anti-G) (A), D25 (anti-F pre-fusion form only) (B), and Palivizumab (anti-F site II reactive with pre-fusion and post-fusion forms) (C) were analyzed for total binding to purified pre-fusion RSV-F (red), post-fusion RSV-F (blue), non-glycosylated RSV-G (black), and glycosylated RSV-G (green) proteins. Total antibody binding is represented in SPR resonance units. (TIF) [file ppat.1005554.s006.tif]
